# Action of Cold on the Lungs

**Published:** 1831-01-01

**Authors:** 


### Action of Cold on the Lungs.

M. Flourens, the celebrated French experimentalist, has been endeavouring to determine the effects of cold and warm air on the lungs. We shall glance at these experiments. Birds were selected as the subjects. A young one was suddenly exposed to a severe and continued cold temperature. A great oppression of the chest was soon evident—the breathing was laborious, the bird refused food and drink, and died in a few hours of acute pneumonia. On dissection the lungs were found to be red and gorged with blood.

Other experiments were made, where the cold was gradually applied, and several times interrupted—in short, the animal was exposed to considerable atmospherical transitions. In these cases, chronic inflammation of the lungs was the result. On dissection the organ was, in some places, hepatized—in others, infiltrated with pus. In a third series of experiments, M. Flourens confined several young chickens in a mild and warm atmosphere—and exposed an equal number, from the same hatch, to all the atmospherical variations of the season. The whole of the former remained free from disease—most of the latter fell victims to pulmonary phthisis. In a fourth set of trials, several young chickens that had been exposed to atmospherical variations, and began to exhibit the signs of pulmonary disease, were placed in localities where the temperature was mild and kept uniform. The greater number of them regained health though very slowly. The lungs of these, on examination, presented some small and flattened vesicles, in which were minute black points. From these experiments the author concludes first, that cold has a direct and special action on the lungs, productive of acute or chronic inflammation, according to its intensity, and the suddenness of its application—secondly, that a mild and uniform heat prevents the invasion of inflammation and of phthisis—and, in many instances, removes these diseases after having commenced.

It is hardly necessary to remark that these experiments, provided they are correct, and the inferences just, only quadrate with an almost universal opinion in the community at large, and even in the medical profession—namely, that pulmonary diseases are chiefly owing to the cold and variable climate of Britain and similarly situated countries—and that the grand means of prevention or cure consist in migrating to warmer skies, or in keeping to a regulated temperature at home. It is probable too, that the opinion so generally diffused, is not without foundation in truth. The difficulty is, to find a climate where the air is mildly warm in the degree of its temperature, and free from sudden vicissitudes. We have perambulated a good deal over the surface of this earth, and few indeed are the localities where this happy combination of mildness and uniformity is to be found! Between the Tropics, we have, no doubt, a steady range of temperature for the greater part of the year; but that range is too high for invalids in general; laying aside the difficulty of the voyage, and the want of comforts when beyond the seas. In those countries on the Continent, so long celebrated for their anti-phthisical influence, there is any thing but a steady range of atmospherical temperature. If we look to fair Italy, we shall perceive that this boasted country is very unfavourably situated for steadiness of climate. With its feet resting against the snow-clad alps, and its head stretching towards the burning shore of Africa, it is alternately exposed to the suffocation of the SIROCCO from the arid sands of Lybia, and to the chilling Tramontane from the Alps or the Apennines. From the relative situation indeed of these two chains of mountains and the African sands, it would not be an exaggeration to say that, almost every breeze, in Italy, comes over a volcano or an iceberg—and consequently we are alternately scorched by the one, and frozen by the other. If it be said that these exorbitant vicissitudes are very *infrequent*, as compared with atmospherical transitions in our own climate: to this we reply that, they are so much the more dangerous. The limitation of the range of atmospheric variation, and the *frequency* of its occurrence, are the very best protections against the deleterious effects of thermometrical, barometrical, and hygrometrical transitions. By this frequency of occurrence, we become *inured* to the atmospherical changes; whereas the long intervals between rapid and intense degrees of heat and cold, drought and moisture, in Italy, deprive us of the protecting influence of habit.

Sir Humphry Davy, who travelled more in the character, and with the qualifications of a philosopher than of a physician, would persuade us that the climate of Italy is a Paradise.

"In the mild climate (says he) of Nice, Naples, or Sicily, where, even in winter, it is possible to enjoy the warmth of the sunshine in the open air beneath palm trees, or amidst the ever-green groves of orange-trees, covered with odorous fruit and sweet-scented leaves, mere existence is a pleasure, and even the pains of disease are sometimes forgotten amidst the balmy influence of Nature; and a series of agreeable and uninterrupted sensations invite to repose and oblivion."*

Yes! but when we come to be startled from this bed of roses by the SIROCCO or the TRAMONTANE, we find, to our cost, that the longer the series of agreeable sensations, the more susceptible do we become to the deleterious influence of the enormous transition of the climate. A female writer

* Consolations of Travel.

has drawn a more accurate picture of the delightful climate of Naples than the late President of the Royal Society.

"In Rome and its surrounding deserts, every thing depicts the death of Nature. In Naples and its environs, all evinces her vigour and activity—*an activity that preys on itself—a feverish vitality that consumes while it brightens. The air is fire, the soil a furnace. Sunbeams bring death! And the earth when struck sends up burning vapours*."*

But, as many months may not elapse before we have an opportunity of more fully exposing our views respecting the effects of an Italian climate on health and disease, we shall drop the subject for the present.

